# Percutaneous Left Atrial Appendage Occlusion Versus Oral Anticoagulation in Atrial Fibrillation: A Systematic Review and Bayesian Meta-analysis

**DOI:** 10.1016/j.jscai.2026.105625

**Published:** 2026-09-10

**Authors:** Amar Rai, Abhinav Kandala, Harroop Bola, James Schurr, Mandeep Singh, Yousif Ahmad, Alexander Fanaroff, Ray Hu, Nitin Malik, David S. Frankel, Timothy Markman, Saman Nazarian, Jay Giri, Tayyab Shah, Ashwin Nathan

**Affiliations:** a Department of Internal Medicine, University of Pennsylvania, Philadelphia, Pennsylvania; b Faculty of Medicine, Imperial College London, London, United Kingdom; c Faculty of Medicine, University College London, London, United Kingdom; d Division of Cardiology, Hospital of the University of Pennsylvania, Philadelphia, Pennsylvania; e Department of Medicine, University of Rochester Medical Center, Rochester, New York; f Division of Cardiology, University of California San Francisco, San Francisco, California

**Keywords:** atrial fibrillation, atrial appendage occlusion, Bayesian meta-analysis, direct oral anticoagulant, left stroke prevention

## Abstract

**Background:**

Conflicting randomized trial results have generated uncertainty regarding percutaneous left atrial appendage occlusion (LAAO) vs oral anticoagulation (OAC) for stroke prevention in atrial fibrillation (AF). Thus, the objective of this study is to estimate the posterior probability that LAAO confers clinically meaningful excess ischemic stroke risk relative to OAC.

**Methods:**

A Bayesian hierarchical meta-analysis was performed of RCT comparing LAAO with OAC in nonvalvular AF. The primary outcome was ischemic stroke, analyzed using Bayesian random-effects Poisson regression under a vague (primary), skeptical, and enthusiastic prior specification.

**Results:**

Six RCT (7004 patients, +18,000 patient-years) were included. Ischemic stroke incidence rate ratio (IRR) was 1.34 (95% credible interval [CrI], 0.91-1.97; *P* [IRR > 1.0] = 94%). The probability of exceeding 0.5 and 1.0 excess events/100 patient-years was 12% and 0.5%, respectively. The effect on any-stroke was smaller (IRR, 1.09; 95% CrI, 0.76-1.56; *P* [IRR > 1.0] = 70%; *P* [absolute rate difference >0.5%/y] = 4.7%), because of a reduction in hemorrhagic stroke (IRR, 0.55; 95% CrI, 0.23-1.18; *P* [IRR < 1.0] = 94.5%). The decrease in hemorrhagic stroke was attenuated among trials using direct oral anticoagulants for medical management (IRR, 0.80; 95% CrI, 0.33-1.75).

**Conclusions:**

In AF, there is a high posterior probability that LAAO is associated with a higher rate of any-stroke, driven by an increase in ischemic stroke relative to OAC. However, the 95% CrI for ischemic stroke crossed unity, and the estimated absolute excess is small. This possible excess must be weighed against a substantial reduction in the risk of hemorrhagic stroke.

## Introduction

Atrial fibrillation (AF) confers a 5-fold increase in ischemic stroke risk, with the left atrial appendage implicated as the thromboembolic source in over 90% of cases.^1^ Percutaneous left atrial appendage occlusion (LAAO) currently carries a class IIb recommendation in both the 2023 ACC/AHA/ACCP/HRS and 2020 ESC AF guidelines for patients with nonvalvular AF who require stroke prevention but in whom long-term oral anticoagulation (OAC) is a concern.^2^, ^3^, ^4^, ^5^ This recommendation was established principally on early trials that compared LAAO with warfarin, a comparator now largely superseded by direct oral anticoagulants (DOAC) as the established standard of care for AF stroke prevention.^6^^,^^7^ DOAC offer superior efficacy-safety profiles relative to vitamin K antagonists (VKA), yet lifelong anticoagulation still carries a 2% to 4% annual major bleeding risk, and real-world discontinuation rates approach 30% to 40% within 2 years.^2^, ^3^, ^4^, ^5^, ^6^, ^7^, ^8^, ^9^

In the contemporary DOAC era, multiple new randomized trials have compared LAAO with modern OAC, yielding conflicting results and employing at times highly variable noninferiority thresholds that complicate cross-trial interpretation.^10^, ^11^, ^12^, ^13^, ^14^ Stroke is the outcome patients most fear and to which they assign the greatest negative weight, with quantitative preference studies demonstrating that many patients regard surviving a disabling stroke as worse than death.^15^ Existing meta-analyses have either predated pivotal contemporary evidence or have prioritized composite end points combining ischemic and hemorrhagic events of different clinical severity, limiting the precision with which events that patients care about most (stroke) can be characterized.^14^^,^^16^^,^^17^

Against this background, an evolving standard of care, genuinely discordant contemporary evidence, and noninferiority margins that vary substantially across trials, a rigorous probabilistic appraisal of the specific effect of LAAO on ischemic stroke risk in absolute terms is needed. We, therefore, conducted a systematic review and Bayesian meta-analysis of all available randomized controlled trials (RCTs) comparing LAAO with OAC.

## Materials and Methods

### Study design and protocol registration

This systematic review and Bayesian meta-analysis was conducted in accordance with PRISMA 2020 guidelines and the Cochrane Handbook for Systematic Reviews of Interventions.^18^^,^^19^ The full protocol was prospectively registered in PROSPERO (CRD420261357391)^20^ prior to initiation of the formal literature search and data extraction.

### Literature search strategy

A comprehensive systematic search was conducted across 5 electronic databases: MEDLINE, Embase, Cochrane Central Register of Controlled Trials, CINAHL, and Scopus, from database inception through April 10th 2026. The full Boolean search string is provided in Supplemental Appendix. Search terms combined MeSH headings and free-text keywords pertaining to AF, LAA occlusion or closure, and OAC. ClinicalTrials.gov and the World Health Organization International Clinical Trials Registry Platform were searched in parallel. No language, date, or geographic restrictions were applied. Citation searching of included studies and relevant systematic reviews was performed to supplement the electronic database search.

### Eligibility criteria

Studies were required to be RCT that enrolled adults aged ≥18 years with documented nonvalvular AF (paroxysmal, persistent, or long-standing) who were candidates for long-term OAC for stroke prevention. The intervention was percutaneous LAAO, using any commercially available or investigational percutaneous occluder within an eligible RCT.

The comparator was OAC (either VKA or DOAC). Given that DOAC have superseded VKA as the recommended first-line therapy in current guidelines, VKA-controlled trials are retained in the primary analysis for completeness but are explicitly isolated in a prespecified sensitivity analysis. Studies were required to report at least 1 prespecified efficacy or safety end point with outcome data presented separately by treatment arm at a minimum follow-up of 12 months. Trials enrolling less than 100 patients were also excluded.

A specific consideration applies to CLOSURE-AF, in which the control group received physician-directed best medical care rather than a protocol-mandated single agent: the majority received a DOAC, approximately 15% received a VKA, and a small proportion received no oral anticoagulant. Because outcomes in that trial were not stratified by the specific agent received, CLOSURE-AF was modeled using its aggregate control arm event counts and person-time as a single, DOAC-predominant comparator node and was, therefore, retained in the DOAC comparator sensitivity analysis.

### Outcome measures

#### Primary outcome

The prespecified primary outcome was ischemic stroke, adjudicated according to the criteria of each individual trial. The primary prespecified metric was the hazard ratio (HR); however, because the majority of included trials reported aggregate event counts and person-time rather than Cox proportional hazards estimates, the incidence rate ratio (IRR) derived from Bayesian hierarchical Poisson regression was used as the primary effect metric.

#### Secondary efficacy outcomes

Prespecified secondary efficacy outcomes included: (1) hemorrhagic stroke or intracranial hemorrhage of any etiology; (2) any-stroke composite combining ischemic and hemorrhagic events within trial; (3) all-cause mortality; (4) cardiovascular mortality; and (5) net clinical benefit composite (cardiovascular death, hemorrhagic stroke, systemic embolism, and nonprocedural major bleeding, harmonized across trials where composite definitions differed).

#### Safety outcomes

Prespecified safety outcomes included: (1) major bleeding (all causes, ISTH-defined or BARC ≥3); (2) nonprocedural major bleeding, extracted separately to isolate the chronic anticoagulation-related safety signal from periprocedural bleeding; (3) clinically relevant nonmajor bleeding (CRNMB); and (4) any nonprocedural bleeding. Device-related thrombus (DRT) was reported descriptively for the LAAO arm only.

### Study selection and data extraction

Retrieved records were imported into Covidence systematic review management software.^21^ Duplicate removal, title/abstract screening, and full-text review were performed by 2 independent reviewers (A.K. and H.B.), with discordant determinations adjudicated by a third reviewer (A.R.) through consensus discussion. Data extraction was performed using a standardized, piloted electronic form. Extracted variables included study identifiers, randomization method, sample size, population demographic characteristics, device type, comparator OAC agent, follow-up duration, and outcome event counts or rates. Where tabular data were insufficient, event counts were cross-verified against published Kaplan-Meier curves using WebPlotDigitizer (version 4.6). Where an eligible RCT published multiple follow-up timepoints, data from the longest available follow-up were used in the primary analysis.

### Quality assessment and risk of bias

Methodological quality and risk of bias were independently assessed by at least 2 reviewers using the revised Cochrane risk of bias tool for randomized trials (RoB-2),^22^ across 5 domains: randomization process; deviations from intended interventions; missing outcome data; outcome measurement; and selection of reported results. Given only 6 eligible RCT, formal funnel plot testing was not performed because of insufficient statistical power to detect small-study effects.

### Statistical analysis

#### Statistical model

We used a Bayesian hierarchical Poisson model with random intercepts and random treatment effects by trial. A posterior probability expresses the probability that a hypothesis is true given the observed data and the specified prior. This quantity is a direct probabilistic statement about the effect itself rather than about the frequency of data under a null hypothesis, and the accompanying 95% credible interval (CrI) denotes the range within which the true effect lies with 95% probability.

For each outcome, the count of events in each arm of each trial was modeled as Poisson distributed with rate parameter λ equal to the product of the arm-specific incidence rate and the patient-time at risk. The treatment effect was parameterized as a log IRR with a normal prior whose mean and variance varied by sensitivity specification.

Three priors were used on the log IRR: vague (normal [0, 10^2^]) as the primary specification; skeptical (normal [0, 0.5^2^]) centered on the null; and enthusiastic (normal [–0.22, 0.5^2^]), centered on a 20% relative risk reduction. Within this framework, a skeptical prior conservatively assumes no difference between LAAO and OAC, requiring robust evidence to demonstrate a treatment effect. Conversely, an enthusiastic prior reflects a baseline expectation of clinical benefit with LAAO. Evaluating the data across skeptical, enthusiastic, and vague prior specifications ensures that the final conclusions are robust to differing initial assumptions.

Models were fit with brms in R using Stan, with 4 chains, 60,000 iterations per chain, and the first 10,000 discarded as warmup, yielding 200,000 postwarmup posterior draws per outcome per prior.^23^^,^^24^ For outcomes, posterior probabilities are reported in the harm and benefit direction: *P* (IRR > threshold) and *P* (IRR < threshold), respectively, which quantifies the probability that LAAO increases ischemic stroke risk, and *P* (absolute rate difference [ARD] > threshold) quantifies the probability that LAAO imposes an absolute excess exceeding the stated threshold.

The absolute risk thresholds of 0.5, 1.0, and 1.5 events per 100 patient-years were prespecified to span the range of effect sizes considered clinically actionable in stroke prevention decision-making. A difference of 0.5 events per 100 patient-years approximates the smallest contrast that patient preference and shared decision-making studies suggest is likely to alter an individual patient's treatment choice, whereas 1.0 and 1.5 events per 100 patient-years represent progressively more decisive differences that would be expected to influence guideline-level recommendations. Anchoring the posterior probabilities to these graded thresholds allows the reader to judge whether a difference between LAAO and OAC exists, and if its magnitude is large enough to be clinically consequential.

#### Sequential Bayesian updating

A prespecified novel analytic element was sequential (cumulative) Bayesian updating, demonstrating how the posterior distribution for ischemic stroke IRR evolved as each trial was incorporated chronologically. Results are presented as evolving posterior density curves and cumulative posterior probability plots.

### Subgroup analyses

Comparator type and device generation subgroups were modeled by separately fitting the hierarchical Poisson model to the subset of trials in each stratum, preserving the same prior structure as the primary analysis. Patient-level subgroups (age, sex, CHA_2_DS_2_-VASc, and AF type) used a 2-stage approach: trial-level subgroup HR extracted from each publication were pooled using a Bayesian random-effects model on the log scale.

### Outcome definitions

Ischemic stroke and hemorrhagic stroke followed each trial’s primary adjudicated definition. The any-stroke composite combined both event types within each trial. Nonprocedural major bleeding excluded events occurring within 7 days of the index procedure (or 30 days where reported) and applied each trial’s ISTH-aligned major bleeding definition.

Net clinical benefit followed the composite definition reported in each trial: typically, death, stroke, systemic embolism, and major bleeding. Where a trial reported multiple composites, we used the one that most closely matched this definition. We extracted the composite outcomes reported directly from each primary trial and did not harmonize them through individual data extraction.

### Sensitivity analyses

The primary analysis was repeated under 3 sensitivity specifications: (1) skeptical prior on the log IRR; (2) restriction to DOAC comparator trials (excluding PROTECT AF and PREVAIL)^6^^,^^7^; and (3) exclusion of OPTION (the postablation enrollment population).^11^

### Software

The analysis was conducted in R 4.3.3 (The R Foundation) using the brms package interfacing with Stan via cmdstanr. Posterior summaries used the posterior package.

### Ethical statement

This study was a systematic review and Bayesian meta-analysis of previously published, deidentified data from randomized controlled trials and did not involve access to individual patient data. Ethics committee approval and individual informed consent were, therefore, not required.

## Results

### Study selection

The systematic search identified 2441 records across all databases. Following automated removal of 5 duplicates, 2436 records underwent title and abstract screening, of which 81 proceeded to full-text review. Six RCT met all prespecified eligibility criteria and were included in the quantitative Bayesian synthesis.^6^^,^^7^^,^^10^, ^11^, ^12^, ^13^ The PRISMA flow diagram is presented in Figure 1.

**Figure 1:**
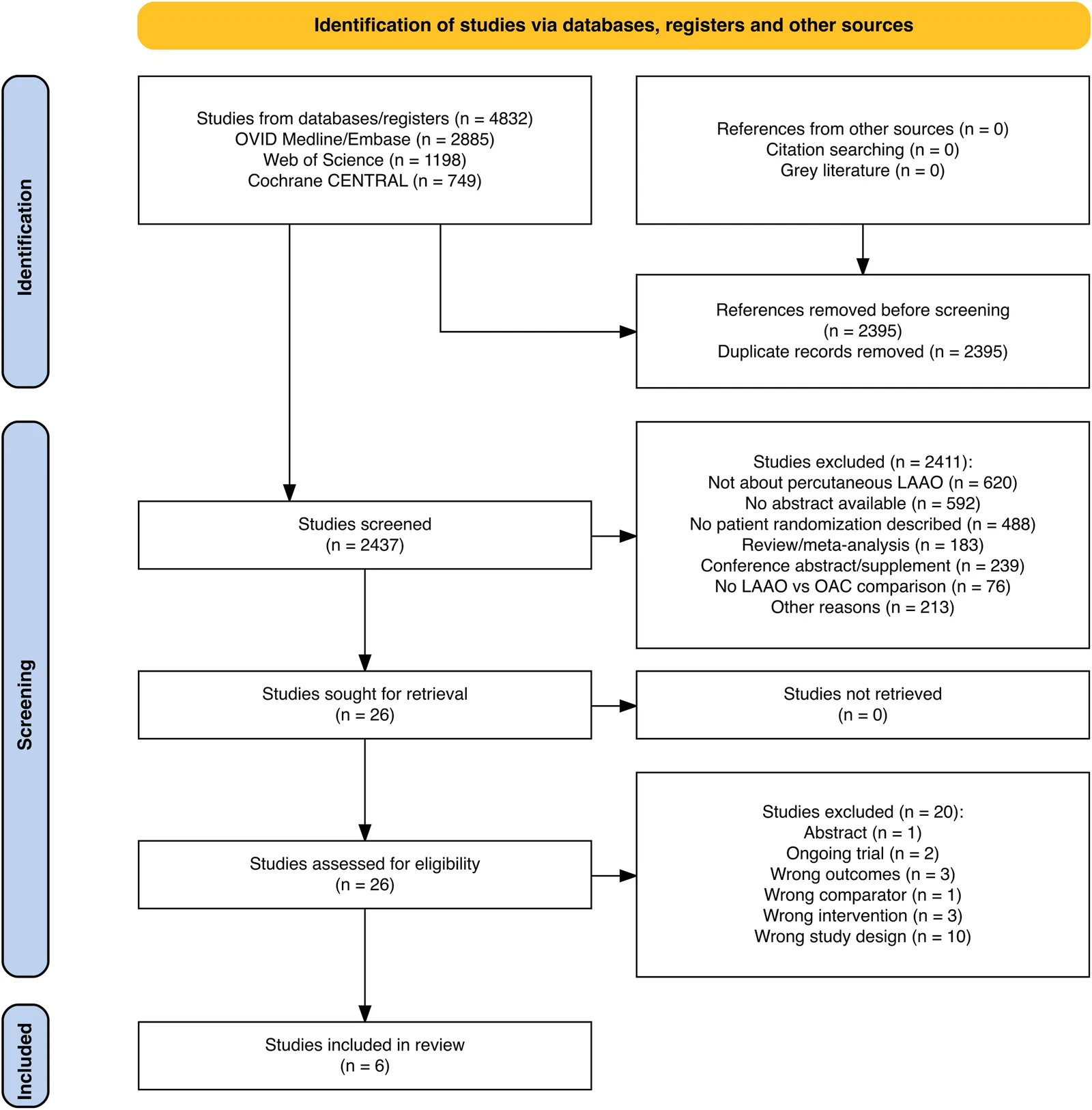
**PRISMA flow diagram.** Records were identified across 5 electronic databases and trial registries. Exclusion reasons at full-text review: wrong study design (n = 10); conference abstract without a corresponding full-text peer-reviewed publication at any follow-up timepoint (n = 1); ongoing trial without published results at any timepoint (n = 2); wrong outcomes (n = 3); wrong comparator (n = 1); wrong intervention (n = 3). Six RCT met all prespecified eligibility criteria and were included. LAAO, left atrial appendage occlusion; OAC, oral anticoagulation.

### Study characteristics

This meta-analysis included 6 RCT (PROTECT AF, PREVAIL, PRAGUE-17, OPTION, CLOSURE-AF, and CHAMPION-AF) comprising 7004 adults with nonvalvular AF eligible for long-term OAC (Table 1).^6^^,^^7^^,^^10^, ^11^, ^12^, ^13^ Patients were randomized to percutaneous LAAO (n = 3681) or OAC (n = 3323), with median follow-up ranging from 1.0 to 3.8 years.^6^^,^^7^^,^^10^, ^11^, ^12^, ^13^ The interventional and comparator strategies reflect the chronological evolution of the field: foundational trials (PROTECT AF, PREVAIL) evaluated the first-generation Watchman device against warfarin, whereas the 4 contemporary trials evaluated modern endocardial occluders (Watchman FLX and AMPLATZER Amulet) predominantly against DOAC.^6^^,^^7^^,^^10^, ^11^, ^12^, ^13^ The risk of bias of each trial is evaluated in Supplemental Figure S1.

**Table 1:** Characteristics of included randomized controlled trials

Study, year	Country/region	N	LAAO arm (n)	OAC arm (n)	LAAO device(s)	Specific OAC agent(s)	Median follow-up, y
PROTECT AF, 2014^6^	USA, Europe	707	463	244	Watchman	Warfarin	3.8
PREVAIL, 2014^7^	USA	407	269	138	Watchman	Warfarin	1
PRAGUE-17, 2020^10^	Czech Republic	402	201	201	Amulet (61%), Watchman (36%), Watchman FLX (3%)	DOAC (apixaban 95.5%)	1.7
OPTION, 2025^11^	Europe	1600	803	797	Watchman FLX	DOAC (95%)/VKA (5%)	3
CLOSURE-AF, 2026^12^	Germany	888	446	442	Watchman/Watchman FLX/Amulet/LAmbre	DOAC or warfarin	3
CHAMPION-AF, 2026^13^	16 Countries	3000	1499	1501	Watchman FLX	DOAC	3

DOAC, direct oral anticoagulant; LAAO, left atrial appendage occlusion; OAC, oral anticoagulation; VKA, vitamin K antagonist.

Baseline demographic characteristic and clinical characteristics were well balanced between the respective treatment arms (Supplemental Table S1). The pooled mean age was 72.3 ± 8.0 years in the LAAO arm and 72.2 ± 8.0 years in the OAC arm, with women comprising 33.4% and 32.7% of the cohorts, respectively. Paroxysmal AF was the predominant rhythm (58.7% LAAO vs 61.3% OAC) among the 4 trials reporting AF subtype. Baseline thromboembolic and bleeding risks were analogous, reflected by a pooled mean CHA_2_DS_2_-VASc score of 3.8 ± 1.4 (LAAO) vs 3.8 ± 1.5 (OAC), and a pooled mean HAS-BLED score of 1.7 ± 1.1 vs 1.6 ± 1.1 across the 4 applicable trials.^10^, ^11^, ^12^, ^13^ A prior stroke or transient ischemic attack was present in 15.5% of patients in both arms.

### Primary outcome: Ischemic stroke

In the primary analysis employing a noninformative prior, LAAO was associated with a posterior median IRR of 1.34 (95% CrI, 0.91-1.97) for ischemic stroke across the 6 included trials (110 events in LAAO vs 72 in OAC; Figure 2; Table 2). The posterior probability of a relative excess in ischemic stroke risk (*P* [IRR > 1.0]) was 94%. Furthermore, the probabilities of exceeding relative risk thresholds of 30% (*P* [IRR > 1.3]) and 50% (*P* [IRR > 1.5]) were 56% and 27%, respectively.

**Figure 2:**
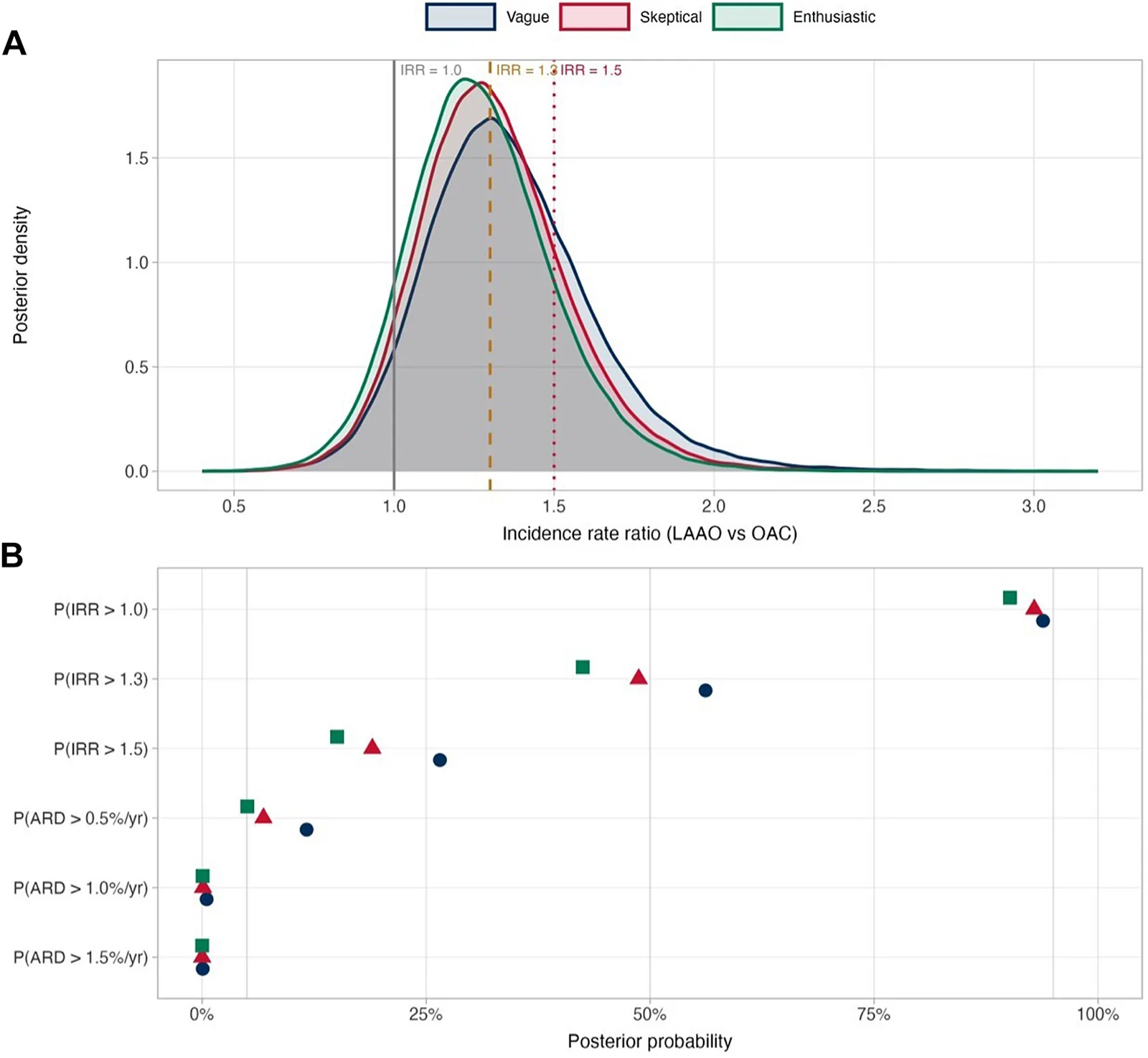
**Posterior probability of ischemic stroke harm with left atrial appendage occlusion (LAAO) compared with oral anticoagulation (OAC).** Bayesian hierarchical Poisson model fit to 6 randomized trials (N = 7004; 200,000 postwarmup draws). Incidence rate ratio (IRR) >1 favors OAC; IRR <1 favors LAAO. Panel (**A**) shows posterior densities of the IRR under 3 priors on the log IRR: vague (normal [0, 10^2^]), skeptical (normal [0, 0.5^2^]), and enthusiastic (normal [–0.22, 0.5^2^]). Panel (**B**) shows posterior probabilities that the IRR or absolute rate difference exceeds clinically meaningful thresholds. ARD thresholds are computed relative to the pooled OAC baseline rate of 0.75 events per 100 PY. ARD, absolute rate difference.

**Table 2:** Bayesian meta-analysis results: LAAO vs OAC in atrial fibrillation

Outcome	Category	k	Events (LAAO/OAC)	Median IRR (95% CrI)	50% CrI	*P* (IRR > 1.0)	*P* (IRR > 1.3)	*P* (IRR > 1.5)	*P* (ARD > 0.5%/y)	*P* (ARD > 1.0%/y)	τ (95% CrI)
Ischemic stroke	Primary outcome	6	110/72	1.34 (0.91-1.97)	1.18-1.51	94%	56%	27%	12%	0.5%	1.17 (1.007-1.936)
Any-stroke (ischemic + hemorrhagic)	Patient-centered composite	6	132/104	1.09 (0.76-1.56)	0.98-1.22	70%	15%	3.7%	4.7%	<1%	1.195 (1.008-1.958)
Hemorrhagic stroke	Secondary: efficacy	5	21/32	0.55 (0.23-1.18)	0.43-0.70	5.5%	1.6%	0.9%	<1%	<1%	1.412 (1.017-4.626)
All-cause mortality^a^	Secondary: efficacy	4	312/286	0.95 (0.66-1.28)	0.87-1.03	33%	2.2%	0.8%	6.6%	1.9%	1.175 (1.008-2.025)
Cardiovascular mortality	Secondary: efficacy	6	153/142	0.92 (0.59-1.36)	0.81-1.04	32%	3.6%	1.2%	2.8%	0.5%	1.332 (1.019-2.406)
Net clinical benefit^b^	Secondary: efficacy	4	410/505	0.84 (0.51-1.33)	0.74-0.94	17%	2.9%	1.3%	9.1%	5.3%	1.377 (1.106-2.721)
Major bleeding (all)	Secondary: safety	5	211/226	0.87 (0.63-1.16)	0.79-0.95	14%	0.8%	<1%	1.5%	<1%	1.184 (1.009-1.826)
Nonprocedural major bleeding	Secondary: safety	4	125/160	0.7 (0.46-0.98)	0.62-0.78	2.1%	<1%	<1%	<1%	<1%	1.168 (1.007-2.013)
CRNMB^c^	Secondary: safety	2	145/303	0.47 (0.22-1.06)	0.41-0.54	2.8%	1.7%	1.3%	2.3%	1.9%	1.252 (1.010-5.332)
Any nonprocedural bleeding^c^	Secondary: safety	2	219/397	0.54 (0.23-1.26)	0.47-0.61	4.2%	2.4%	1.8%	3.5%	2.9%	1.289 (1.013-5.842)
Hemorrhagic stroke (DOAC comparator only)	Sensitivity: efficacy	4	18/22	0.8 (0.33-1.75)	0.61-1.03	27%	10%	5.3%	<1%	<1%	1.257 (1.010-3.493)

Events columns show total LAAO and OAC events summed across contributing trials. *P* (IRR > x) is the posterior probability that the rate ratio exceeds x. *P* (ARD > y%/y) is the posterior probability that LAAO worsens the outcome rate by at least y percentage points per year (positive ARD favors OAC for harm-direction outcomes; for benefit direction outcomes, the same column shows the probability of net harm at that magnitude). All outcomes from the primary analysis and additional patient-centered and sensitivity outcomes. Bayesian hierarchical Poisson model with vague prior (normal [0, 10^2^] on the log IRR); 200,000 postwarmup draws.

ARD, absolute rate difference; CrI, credible interval; CRNMB, clinically relevant nonmajor bleeding; DOAC, direct oral anticoagulant; IRR, incidence rate ratio (LAAO vs OAC); k, number of trials contributing to the row; LAAO, left atrial appendage occlusion; OAC, oral anticoagulation; τ, between-trial SD on the log IRR.

a PRAGUE-17 excluded from all-cause mortality analysis (event counts not extractable from publication).

b Net clinical benefit composite definition varies by trial: PROTECT AF (stroke + SE + CV death); CLOSURE-AF and CHAMPION-AF additionally include nonprocedural major bleeding.

c Derived from 2 trials only (OPTION, CHAMPION-AF); between-trial heterogeneity not reliably estimable. Presented as exploratory.

When evaluated in absolute terms, the posterior probability of an excess exceeding 0.5 events/100 PY was 12%, while the probability of exceeding 1.0 event/100 PY was only 0.5%. Applying the 34% relative increase to the pooled baseline ischemic stroke incidence in the OAC arm (approximately 0.75 events/100 PY) yields an estimated absolute difference of roughly 0.26 events/100 PY.

In context, this means that among 1000 patients followed for 1 year, the estimated posterior median excess attributable to LAAO rather than OAC is approximately 2.6 additional ischemic strokes.

### Sensitivity and subgroup analyses: Ischemic stroke

Sensitivity analyses (Table 3) confirmed robustness across all prior specifications. Under the skeptical prior, the posterior median IRR was 1.29 (95% CrI, 0.90-1.82; *P* [IRR > 1.0] = 92.8%; *P* [ARD > 0.5/100 PY] = 6.9%). Under the enthusiastic prior, the posterior median was 1.26 (95% CrI, 0.87-1.77; *P* [IRR > 1.0] = 90.4%; *P* [ARD > 0.5/100 PY] = 5.0%). Restriction to the 4 contemporary DOAC comparator trials only (SA2) yielded a consistent posterior median IRR of 1.30 (95% CrI, 0.78-2.12; *P* [IRR > 1.0] = 87.6%; *P* [ARD >0.5/100 PY] = 13.3%), confirming that the ischemic stroke signal is reproducible against modern OAC. Exclusion of OPTION, which enrolled a postablation population with lower baseline ischemic risk (SA3), resulted in a higher posterior estimate (IRR, 1.41; 95% CrI, 0.94-2.14; *P* [IRR > 1.0] = 95.4%). Across all specifications, the posterior median IRR ranged from 1.26 to 1.41, and the posterior probability of any relative excess ischemic stroke remained 90.4% to 95.4%. Sequential Bayesian updating demonstrated similar results (Supplemental Figure S2).

**Table 3:** Sensitivity analysis: Ischemic stroke (primary outcome). Effect of prior specification and trial exclusion on posterior estimates

Model specification	k	Posterior median IRR	95% CrI	*P* (IRR > 1.0)	*P* (IRR > 1.3)	*P* (ARD^a^ > 0.5%/y)
Primary, vague prior (6 trials)	6	1.34	0.91-1.97	93.9%	56.4%	11.6%
Primary, skeptical prior (6 trials)	6	1.29	0.90-1.82	92.8%	48.6%	6.9%
Primary, enthusiastic prior (6 trials)	6	1.26	0.87-1.77	90.4%	42.4%	5.0%
SA2^b^: DOAC comparator only (4 trials)	4	1.30	0.78-2.12	87.6%	50.4%	13.3%
SA3^c^: Exclude OPTION (5 trials)	5	1.41	0.94-2.14	95.4%	66.1%	19.3%

ARD, absolute rate difference; CrI, credible interval; DOAC, direct oral anticoagulant; IRR, incidence rate ratio; k, number of trials.

Bold row = primary prespecified analysis. Shaded rows = alternate specifications.

a ARD threshold computed relative to pooled oral anticoagulation ischemic stroke baseline rate (0.75 events/100 patient-years).

b SA2 = excludes vitamin K antagonist comparator trials (PROTECT AF, PREVAIL); isolates the contemporary left atrial appendage occlusion vs DOAC comparison.

c SA3 = excludes OPTION (postablation enrollment population); OPTION is included in the primary analysis.

Subgroup analyses for ischemic stroke are presented in Figure 3. The effect was consistent across all prespecified subgroups: comparator type (VKA vs DOAC), device generation, age, sex, and CHA_2_DS_2_-VASc stratum, with LAAO associated with a numerically higher posterior median IRR in all subgroups.

**Figure 3:**
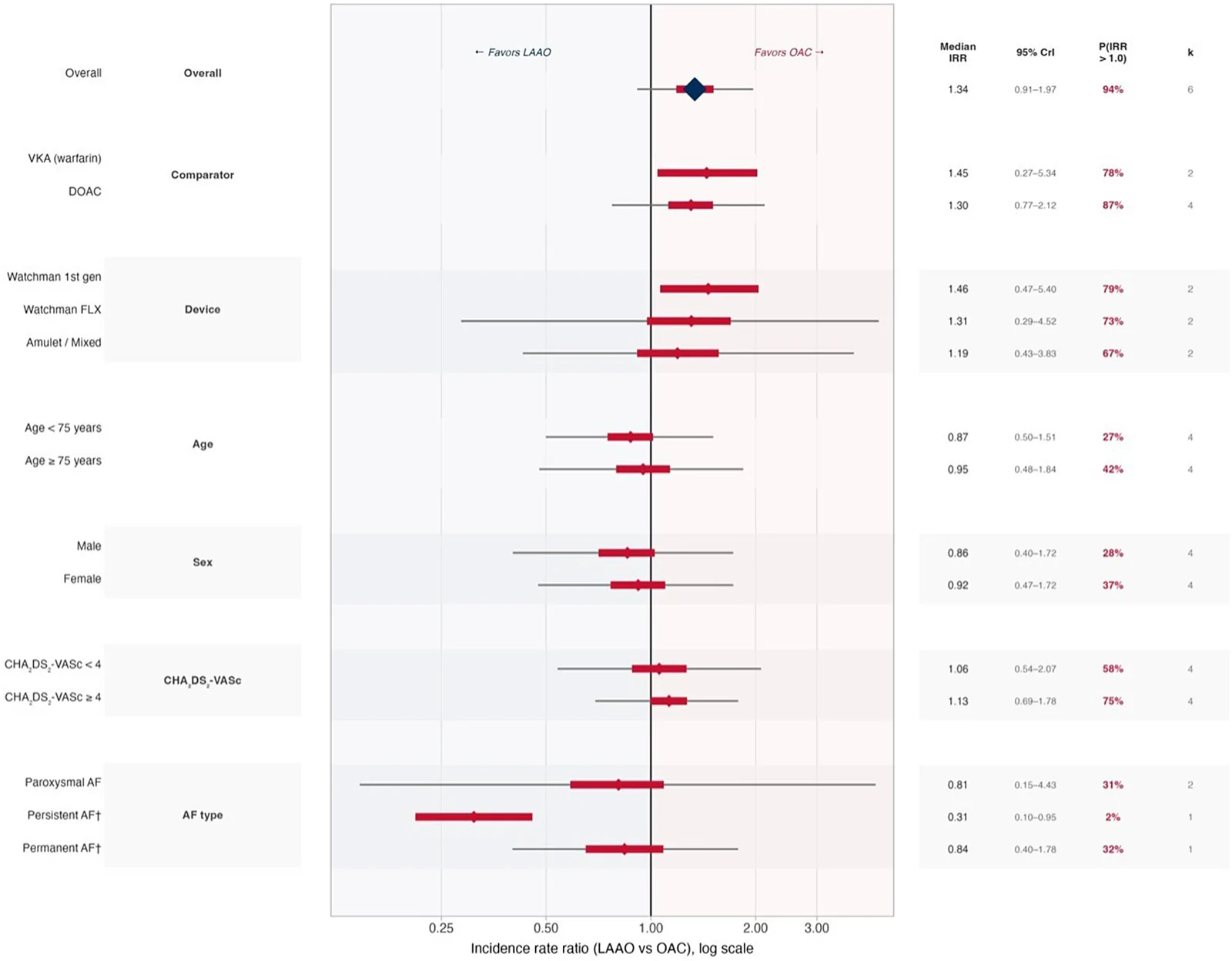
**Subgroup analysis of the LAAO-vs-OAC ischemic stroke incidence rate ratio (IRR).** Diamonds represent posterior median IRR; thick horizontal bars represent 50% credible intervals (CrI); thin horizontal bars represent 95% CrI. The overall estimate is highlighted as a navy diamond. Bayesian hierarchical meta-analysis used a vague prior (normal [0, 10^2^] on the log IRR). Subgroups by comparator type and device generation use separate Bayesian Poisson models fit to trial-level event counts. Subgroups by age, sex, CHA_2_DS_2_-VASc, and atrial fibrillation (AF) type use 2-stage Bayesian pooling of reported trial-level subgroup hazard ratios. Comparator, device, and CHA_2_DS_2_-VASc subgroups were prespecified per PROSPERO. Single-trial rows marked †(persistent AF, permanent AF) are from CHAMPION-AF only and are not pooled. *P* (IRR > 1.0) is the posterior probability of harm. k denotes the number of trials contributing to each row. DOAC, direct oral anticoagulant; LAAO, left atrial appendage occlusion; OAC, oral anticoagulation; VKA, vitamin K antagonist.

### Secondary outcomes

Full secondary outcome results are presented in Table 2. Prespecified threshold posterior probability curves for primary and secondary outcomes are presented in Figure 4 (relative scale) and Figure 5 (absolute scale) for direct comparisons. For hemorrhagic stroke (5 trials; 21 LAAO vs 32 OAC events), LAAO was associated with a substantial posterior reduction: posterior median IRR 0.55 (95% CrI, 0.23-1.18; *P* [IRR < 1.0] = 94.5%). In absolute terms, the posterior median corresponds to a reduction of approximately 0.15 events per 100 PY against a pooled OAC arm hemorrhagic stroke rate of 0.34%/y. When restricted to DOAC comparator trials only (4 trials; 18 LAAO vs 22 OAC events), the hemorrhagic stroke advantage of LAAO attenuated substantially (IRR, 0.80, 95% CrI, 0.33-1.75; *P* [IRR < 1.0] = 73%).

**Figure 4:**
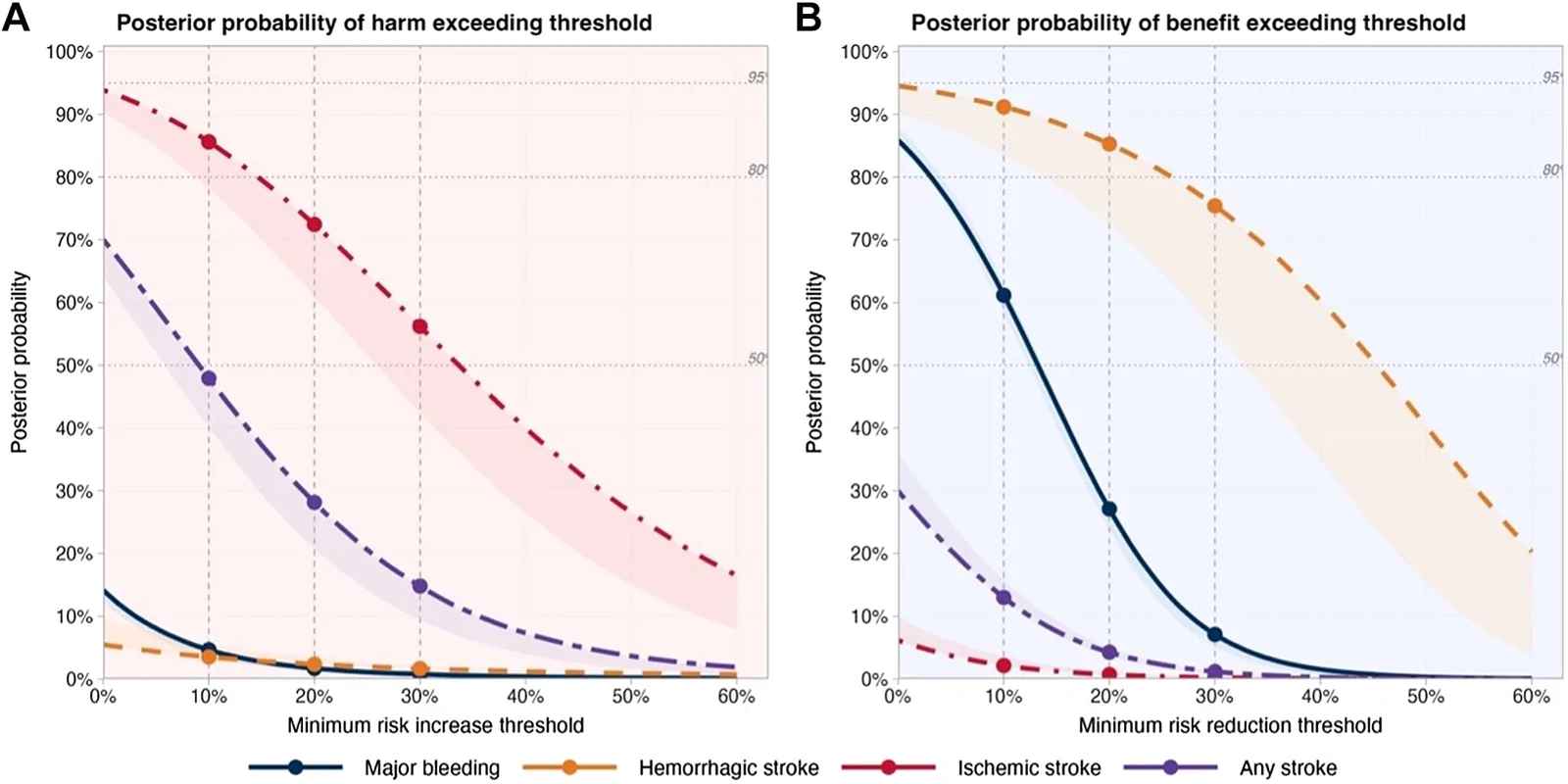
**Posterior probability that left atrial appendage occlusion (LAAO) crosses clinically meaningful thresholds of harm and benefit, by outcome.** Curves show the posterior probability that the absolute rate difference between LAAO and oral anticoagulation exceeds a given magnitude, plotted as a function of that magnitude. Primary curves use a vague prior on the log IRR (normal [0, 10^2^]). Shaded bands span the range across vague, skeptical (normal [0, 0.5^2^]), and enthusiastic (normal [–0.22, 0.5^2^]) prior specifications. (**A**) (Harm): probability LAAO worsens the outcome by at least the indicated amount. Ischemic stroke and the patient-centered any-stroke composite start high (strong harm signal); bleeding curves start near 0. (**B**) (Benefit): probability LAAO improves the outcome by at least the indicated amount. Bleeding curves start high (strong benefit signal); ischemic stroke starts near 0. Reference lines mark posterior probabilities of 50%, 80%, and 95%. Vertical reference lines mark thresholds of 10%, 20%, and 30% absolute rate change. Trials included: PROTECT AF, PREVAIL, PRAGUE-17, OPTION, CLOSURE-AF, and CHAMPION-AF.

**Figure 5:**
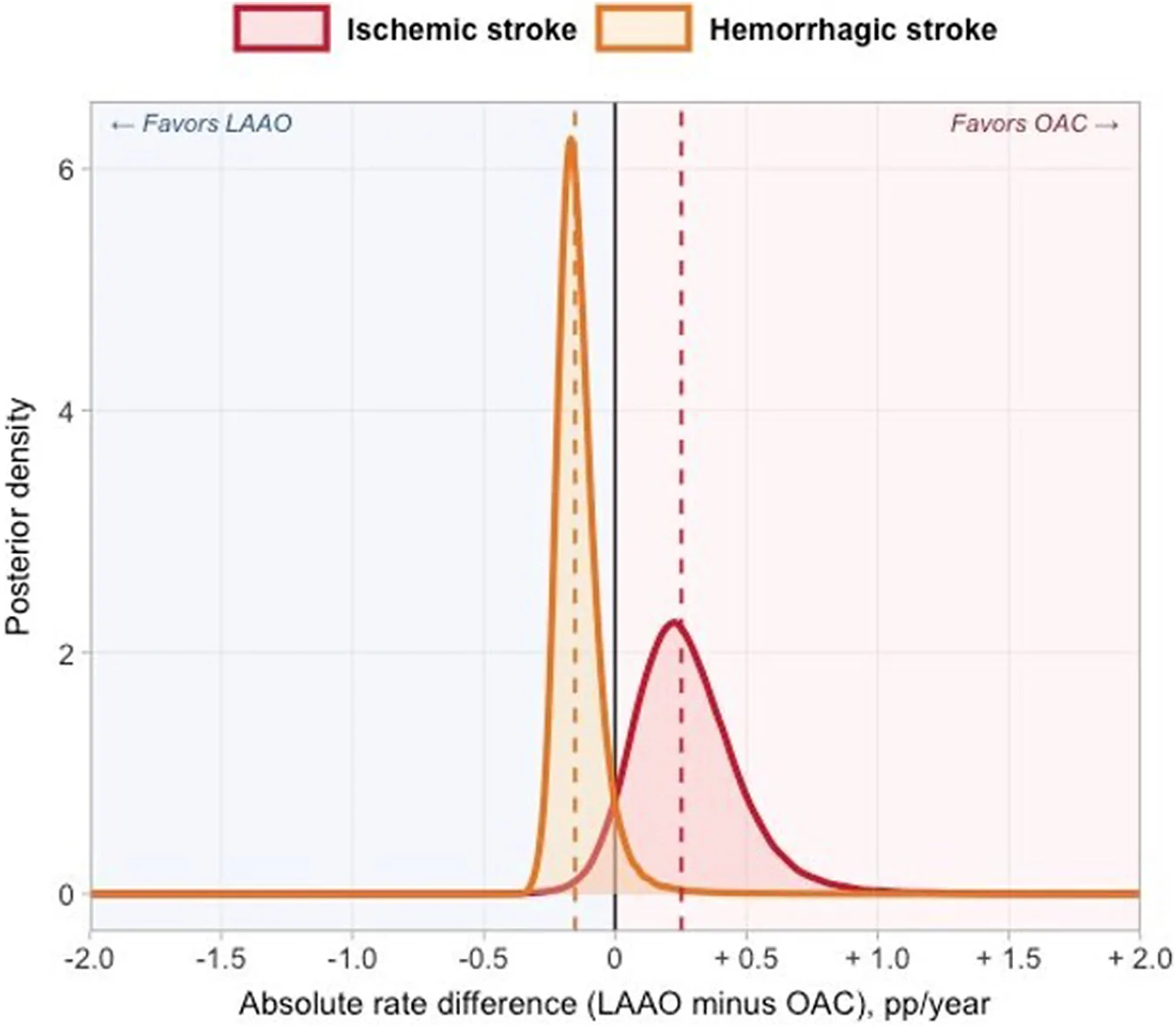
**Posterior densities of the absolute rate difference (left atrial appendage occlusion [LAAO] minus oral anticoagulation [OAC]) for ischemic stroke and hemorrhagic stroke.** Densities are plotted on a percentage points per year scale. Negative values favor LAAO; positive values favor OAC. Vertical dashed lines mark posterior medians; shaded regions encode the directional regions of effect. The ischemic stroke distribution (red) sits to the right of 0 with a posterior median near +0.25 pp/y (favoring OAC); the hemorrhagic stroke distribution (orange) sits to the left of 0 with a posterior median near –0.15 pp/y (favoring LAAO). The absolute magnitudes of both effects are small relative to the spread of plausible values, and the densities overlap at 0. Method: Bayesian hierarchical Poisson model with vague prior (normal [0, 10^2^] on the log IRR); 200,000 postwarmup draws. Absolute rate differences are computed relative to pooled OAC baseline rates of 0.75%/y (ischemic stroke) and 0.34%/y (hemorrhagic stroke). IRR, incidence rate ratio; pp, percentage points.

The any-stroke composite (ischemic plus hemorrhagic events within trial; 6 trials; 132 LAAO vs 104 OAC events) yielded a posterior median IRR of 1.09 (95% CrI, 0.76-1.56; *P* [IRR > 1.0] = 70%). The bidirectional posterior probability curves for this outcome are presented in Supplemental Figure S3.

All-cause mortality was available from 4 trials (312 LAAO vs 286 OAC events) and was similar between groups: posterior median IRR of 0.95 (95% CrI, 0.66-1.28; *P* [IRR < 1.0] = 67%). The posterior probability of ≥0.5 events/100 PY absolute excess in all-cause mortality was 6.6%. Cardiovascular mortality was likewise similar across 6 trials (153 LAAO vs 142 OAC events): IRR 0.92 (95% CrI, 0.59-1.36; *P* [IRR < 1.0] = 68%). The prespecified exploratory net clinical benefit composite (4 trials; 410 LAAO vs 505 OAC events) directionally favored LAAO: IRR 0.84 (95% CrI, 0.51-1.33; *P* [IRR < 1.0] = 83%), driven by the bleeding avoidance signal once the periprocedural period has passed.

All-cause major bleeding was lower in the LAAO arm (5 trials; 211 LAAO vs 226 OAC events; IRR, 0.87; 95% CrI, 0.63-1.16; *P* [IRR < 1.0] = 86%). Similarly, nonprocedural major bleeding was lower in the LAAO arm: IRR 0.70 (95% CrI, 0.46-0.98; *P* [IRR < 1.0] = 97.9%; 125 LAAO vs 160 OAC events across 4 trials). CRNMB was only available in 2 trials, which demonstrated a posterior median IRR was 0.47 (95% CrI, 0.22-1.06; *P* [IRR < 1.0] = 97.2%). Any nonprocedural bleeding similarly favored LAAO in those same 2 trials (219 LAAO vs 397 OAC events): IRR 0.54 (95% CrI, 0.23-1.26; *P* [IRR < 1.0] = 95.8%; exploratory). This end point was not consistently reported in trials and thus removed from analysis.

### DRT

Device-related thrombus was reported descriptively in the LAAO arm across the included trials that recorded this outcome and, when pooled, occurred in 3.57% of device recipients. DRT rates were numerically higher in the earlier warfarin-controlled trials than in the contemporary DOAC comparator trials, consistent with iterative refinements in device design, implantation technique, and postimplantation antithrombotic protocols. Because DRT is a plausible contributor to the observed ischemic stroke signal, it is further explored in the discussion section.

## Discussion

### Principal findings

This systematic review and Bayesian meta-analysis synthesizes the totality of randomized evidence comparing percutaneous LAAO with OAC in nonvalvular AF, encompassing 6 RCT, 7004 patients, and over 18,000 PY of follow-up. Three central conclusions emerge. First, LAAO carries a high posterior probability of a numerically higher ischemic stroke rate than OAC, but the absolute excess is small given low event rates in the AF population in contemporary practice and is unlikely to exceed thresholds of clinical meaningfulness. Second, LAAO is associated with a high posterior probability of reduced nonprocedural major bleeding and a directionally favorable net clinical benefit, indicating that its stroke and bleeding effects must be weighed jointly rather than in isolation. Third, the hemorrhagic stroke advantage of LAAO observed against warfarin attenuates substantially when the analysis is restricted to DOAC comparator trials, the comparison most relevant to contemporary clinical practice, as detailed in the Central Illustration. Holistically, these data support individualized decision-making predicated on the patient’s own stroke-bleeding risk profile, procedural candidacy, and values, rather than a strategy of blanket adoption or blanket restriction of LAAO.

**Central Illustration:**
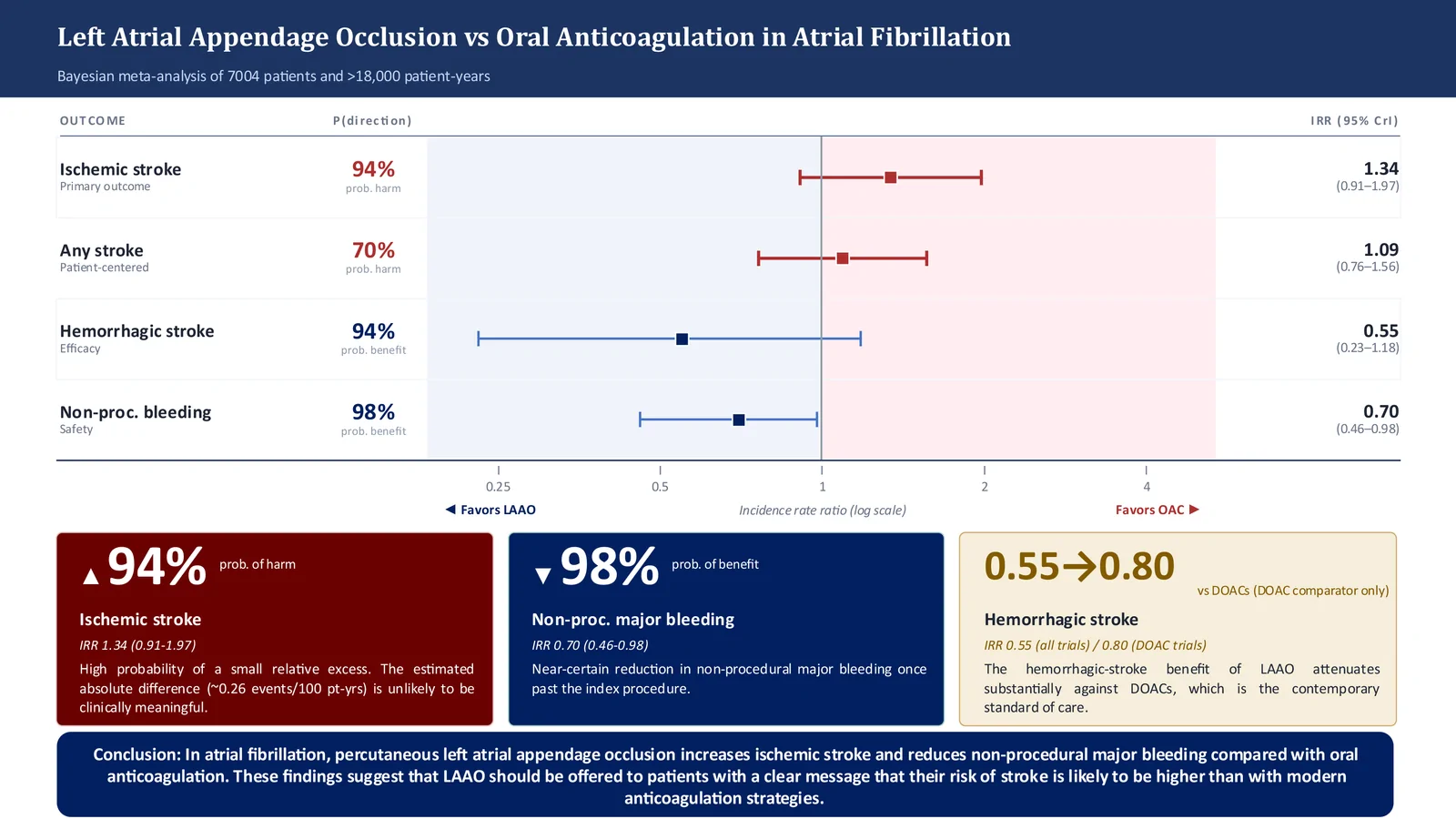
Left atrial appendage occlusion (LAAO) vs oral anticoagulation in atrial fibrillation. CrI, credible interval; DOAC, direct oral anticoagulant; IRR, incidence rate ratio; OAC, oral anticoagulation.

### Contextualizing the ischemic stroke signal

The central tension in interpreting these results lies in reconciling a relative signal with its absolute implications. Across all 6 RCT, the posterior median IRR for ischemic stroke was 1.34, with a 94% posterior probability of directional excess with LAAO. Notably, the 95% CI crossed the line of no significance, indicating that a null or even mildly protective effect for LAAO may remain.^23^, ^24^, ^25^ However, when contextualized against the low baseline ischemic stroke rates observed in both arms across these trials (pooled OAC rate approximately 0.75 events per 100 PY), a 34% relative excess translates to less than 0.3 additional ischemic strokes per 100 PY, which is a magnitude that falls well below thresholds typically considered clinically meaningful in shared decision-making.^23^^,^^25^ This finding has direct relevance to the current clinical debate surrounding LAAO, which has been dominated by relative effect estimates and dichotomous significance testing. The Bayesian framework applied here permits the more clinically intuitive probabilistic statement that, across the totality of randomized evidence, LAAO is unlikely to be associated with a meaningfully higher rate of ischemic stroke than continued OAC therapy.

These estimates were robust across all prespecified sensitivity analyses. Critically, restriction to contemporary DOAC comparator trials only yielded a similar estimate (IRR, 1.30; 95% CrI, 0.78-2.12), confirming that the signal is not an artifact of the historical warfarin-controlled data. Sequential Bayesian updating demonstrated that the posterior progressively concentrated above unity as DOAC era trials entered the model, with the contemporary evidence base, not the warfarin-era foundational trials, driving the current estimate. This has direct implications for guideline interpretation: the class IIb indication for LAAO, established principally in PROTECT AF and PREVAIL, requires recontextualization considering the expanded randomized evidence against modern anticoagulation.^3^^,^^4^^,^^10^^,^^13^

To the best of our knowledge, this is the first meta-analysis to incorporate data from all 6 RCT, including both CLOSURE-AF and CHAMPION-AF, which together contributed 3912 patients, and to apply a fully Bayesian framework with prespecified probability thresholds, sequential updating, and formal prior sensitivity analysis to this clinical question.^12^^,^^13^^,^^23^^,^^24^ Prior frequentist meta-analyses, including those by Madhavan et al^16^ and Lerman et al,^17^ provided valuable initial syntheses but were limited to 4 or fewer trials and relied on dichotomous significance testing, which is poorly suited to the modest sample sizes and heterogeneous comparators that characterize this evidence base.

These findings should inform clinical decision-making and patient discussions in a way that prior analyses have not.^16^^,^^17^ Ischemic stroke is the primary outcome of concern to patients with AF.^15^ Data from patient preference studies demonstrate that patients assign equal or greater importance to avoiding stroke than to avoiding death itself. Stolker et al^25^ reported that cardiovascular patients weighted stroke equivalently to death (mean end point ratio 1.08 relative to death), substantially exceeding the weight assigned to bleeding outcomes. Our analysis directly addresses this priority outcome, as the probability that LAAO causes a clinically meaningful absolute increase in the outcome patients fear most is low.

### Hemorrhagic stroke

Left atrial appendage occlusion was associated with 94.5% posterior probability of reduced hemorrhagic stroke, a reduction that carries disproportionate clinical weight given the 40% to 50% case fatality rate of this stroke subtype, which is approximately 3 to 4 times higher than that of ischemic stroke.^26^^,^^27^ This contributes to the mortality equivalence observed across the trials despite the ischemic stroke signal.

However, 2 aspects affect this finding. First, the absolute magnitude of this reduction is constrained by the low baseline hemorrhagic stroke rate in the OAC arm, with the estimated posterior median absolute reduction being approximately 0.15 events per 100 PY. Secondly, and in relation to clinical practice, when the analysis is restricted to DOAC comparator trials, the hemorrhagic stroke IRR attenuates markedly to 0.80 (95% CrI, 0.33-1.75; *P* [IRR < 1.0] = 73%), compared with 0.55 in the all-trials analysis, reflecting the established hemorrhagic stroke advantage of DOAC over warfarin. Thus, clinicians should not assume that the hemorrhagic stroke benefit observed against warfarin translates directly to DOAC-treated populations. Hemorrhagic stroke contributes to the mortality equivalence observed despite the ischemic stroke signal.^26^^,^^27^

### The patient-centered any-stroke composite

The any-stroke composite (ischemic plus hemorrhagic events; IRR, 1.09; 95% CrI, 0.76-1.56; *P* [IRR < 1.0] = 30%) provides the most transparent single summary of stroke risk. The attenuation from the ischemic stroke-only estimate reflects partial offset by hemorrhagic stroke reduction, but this offset is substantially smaller against DOAC comparators than all-trials analysis implies. The probability of a clinically meaningful absolute any-stroke excess exceeding 0.5 events per 100 PY was only 4.7%, suggesting that when stroke is considered as a unified patient-centered outcome, the likelihood of meaningful net harm from LAAO is low, but the likelihood of meaningful net benefit is similarly modest in the contemporary DOAC era.

### The discordance between CLOSURE-AF and CHAMPION-AF

The apparent contradiction between CLOSURE-AF and CHAMPION-AF is largely explicable by differences in enrolled populations and trial design.^12^^,^^13^ CLOSURE-AF enrolled an older (mean age 78 years), higher-risk population (mean CHA_2_DS_2_-VASc 5.2, HAS-BLED 3.1) in which competing risks of nonstroke mortality are substantial.^12^ In this population, the procedural hazard of LAAO, which is front-loaded, faces a shortened time horizon over which the downstream benefits of anticoagulation avoidance can accumulate. CLOSURE-AF also permitted mixed comparator strategies, utilized multiple device platforms, and employed restricted mean survival time as its primary analytic framework.^12^

CHAMPION-AF, by contrast, enrolled a younger (mean age, 72 years), moderate-risk population (mean CHA_2_DS_2_-VASc 3.5, HAS-BLED 1.3) with longer expected survival, used the Watchman FLX exclusively, and mandated a DOAC comparator.^13^ Despite these differences, the absolute ischemic stroke rates in both trials were low, and when synthesized within our Bayesian framework alongside the 4 other RCT, the overall posterior probability of clinically meaningful ischemic stroke harm remained low regardless of the contribution of either trial, highlighting that the apparent discordance reflects population selection and analytic design, not device-level instability. Importantly, it signals that patient selection criteria will determine the real-world benefit-risk ratio of LAAO at a population level more than device performance alone.

### Bleeding outcomes

Nonprocedural major bleeding was reduced by 30% with LAAO, with a 97.9% posterior probability of benefit. CRNMB showed a directionally large reduction, and any nonprocedural bleeding was similarly favored. These findings are consistent with the primary safety end points of CHAMPION-AF and OPTION.^11^^,^^13^ When all bleeding events, including procedural complications, were analyzed, the treatment effect was attenuated, reflecting the inherent periprocedural hazard of any interventional procedure.

The primary safety analyses in this systematic review and Bayesian meta-analysis, consistent with the prespecified framework of the majority of included trials, focus on nonprocedural bleeding. However, procedural bleeding risk is a patient-relevant hazard that must be incorporated into any shared decision-making framework. Across the included trials, periprocedural complication rates ranged from 2.7% to 8.7%, with the majority attributable to adjunctive antithrombotic therapy rather than device failure.^6^^,^^13^ When all bleeding, including procedural events, was analyzed, the treatment effect was weakened (IRR, 0.87; 95% CrI, 0.63-1.16; *P* [IRR < 1.0] = 86%), demonstrating that the bleeding benefit attributed to LAAO is dependent on surviving a procedure that itself carries early hemorrhagic risk. Clinicians may incorporate this framing into discussions with patients, presenting the decision as a trade-off between upfront procedural risk and downstream anticoagulation-related bleeding avoidance.

Taken together, these bleeding data must be interpreted cautiously within the framework of net clinical benefit. Composites that weight nonprocedural bleeding equivalently with ischemic stroke do not reflect patient preferences. Stolker et al^25^ demonstrated that patients regard these outcomes as fundamentally different in severity. Although the net clinical benefit composite directionally favored LAAO (IRR, 0.84; 95% CrI, 0.51-1.33; *P* [IRR < 1.0] = 83%), the more informative output of this analysis is the probability distribution for ischemic stroke itself, and the demonstration that the absolute excess is small.

### Limitations

This analysis relies on aggregate trial-level data, precluding granular patient-level covariate adjustment. Open-label designs introduce potential ascertainment bias, though blinded adjudication committees mitigated this risk across all-trials. Pooling DOAC into a single comparator node assumes class-effect equivalence that may not hold across medications or dose-reduced regimens, given that apixaban comprised the majority of DOAC exposure.^10^^,^^11^ CLOSURE-AF's use of restricted mean survival time rather than HR required methodological accommodation in the pooled analysis.^12^ Finally, unmodeled competing risks of death and variable follow-up durations limit definitive conclusions regarding very long-term outcomes.

The included trials also differed substantially with respect to comparator therapy, device generation, enrolled populations, and primary analytic framework. This clinical heterogeneity is quantified by the between-trial SD, which is reported for every outcome in Table 2. This is explicitly propagated into the width of the posterior CrI by the random-effects structure of the hierarchical model, so that the reported uncertainty already incorporates cross-trial variability rather than assuming a single common effect.

Nonetheless, the modest number of trials limits the precision with which this heterogeneity can be estimated, and residual variation not fully captured by the modeled random-effects may influence the pooled estimates. The prespecified subgroup and sensitivity analyses by comparator, device generation, and population were designed to investigate this possibility and yielded directionally consistent results.

### Future directions and clinical imperatives

The observed 3.57% pooled DRT rate and the persistent ischemic stroke signal across sensitivity analyses make standardization of postimplantation antithrombotic therapy the most pressing near-term research priority. Mechanistically, DRT offers a biologically plausible explanation for the directional excess in ischemic stroke observed with LAAO, because thrombus forming on the atrial-facing surface of the occluder can embolize prior to endothelialization, and thus, the incomplete, variable-duration antithrombotic regimens used across the included trials may have heightened this hazard.

The ongoing CATALYST trial, evaluating the Amulet device against OAC with a protocol-specified antithrombotic regimen, is expected to provide critical data in this regard.^28^ Patient selection criteria represent the most important determinant of LAAO's real-world benefit-risk ratio. The current evidence base has been built largely in patients with moderate stroke and low-to-moderate bleeding risk. High-risk populations with the strongest mechanistic rationale for LAAO remain underrepresented in randomized evidence. Patients with advanced chronic kidney disease and end-stage renal disease face a convergence of elevated stroke risk, markedly increased OAC-related bleeding risk, and impaired DOAC pharmacokinetics that collectively constitute a compelling case for a nonpharmacological stroke prevention strategy.^29^, ^30^, ^31^, ^32^ Real-world registry data from end-stage renal disease patients suggest a more favorable composite outcome with LAAO compared with warfarin or apixaban^33^; procedural success rates appear comparable to non–chronic kidney disease populations in observational series.^34^ Dedicated randomized evaluation in this population is urgently needed and remains one of the most consequential gaps in the current evidence base.

Finally, the nearly 4-fold variation in serious procedural complication rates across these trials (2.2%-8.6%) highlights the role of operator experience, device generation, and institutional volume in determining real-world outcomes. As LAAO expands to lower-volume programs, the front-loaded procedural risks, which must be weighed against the long-term benefits of avoiding anticoagulation, will likely exceed what trial data from high-volume centers of excellence suggest. Therefore, prospective standardization of imaging-guided protocols and volume-based training metrics is essential to ensure that trial-level safety translates to broader clinical practice. Ultimately, the foundational evidence base has matured. The clinical challenge for the field is no longer establishing whether LAAO works, but rather definitively delineating for whom it works best.

## Conclusion

In this systematic review and Bayesian meta-analysis of 6 RCT (7004 patients), percutaneous LAAO demonstrated a high probability of increased relative ischemic stroke risk compared with OAC, though the absolute excess is small and unlikely to exceed thresholds of clinical meaningfulness under contemporary event rates. This ischemic stroke signal must be interpreted alongside a near-certainty of reduced nonprocedural major bleeding and a directional reduction in all-cause stroke. The hemorrhagic stroke advantage of LAAO, substantial against warfarin, is considerably attenuated against DOAC, the standard most relevant to contemporary clinical practice. These data do not support LAAO as a universal alternative to OAC, but they provide a rigorous probabilistic framework for identifying patients in whom the bleeding-stroke trade-off favors a device strategy. Patients considering LAAO should be counseled explicitly that their ischemic stroke risk is likely to be somewhat higher than with continued modern anticoagulation, that their long-term bleeding risk will be substantially lower, and that a real procedural hazard exists that must be factored into any individualized decision.

## CRediT authorship contribution statement

**Amar Rai:** Writing – review & editing, Writing – original draft, Visualization, Validation, Supervision, Software, Resources, Project administration, Methodology, Investigation, Funding acquisition, Formal analysis, Data curation, Conceptualization. **Abhinav Kandala:** Writing – review & editing, Writing – original draft, Visualization, Validation, Software, Project administration, Methodology, Investigation, Formal analysis, Data curation, Conceptualization. **Harroop Bola:** Validation, Methodology, Formal analysis, Data curation, Conceptualization. **James Schurr:** Methodology, Formal analysis, Data curation. **Mandeep Singh:** Writing – original draft. **Yousif Ahmad:** Supervision, Conceptualization. **Alexander Fanaroff:** Supervision, Conceptualization. **Ray Hu:** Supervision, Conceptualization. **Nitin Malik:** Supervision, Conceptualization. **David S. Frankel:** Supervision, Conceptualization. **Timothy Markman:** Supervision, Conceptualization. **Saman Nazarian:** Supervision, Conceptualization. **Jay Giri:** Supervision. **Tayyab Shah:** Supervision, Conceptualization. **Ashwin Nathan:** Writing – original draft, Supervision.

## Declaration of competing interest

Tayyab Shah has received speaking honoraria from Johnson & Johnson. Timothy Markman has received consulting fees and lecture honoraria from Boston Scientific, Abbott Medical, and Medtronic. Ashwin Nathan has received research funding from Boston Scientific and consulting fees from Edwards Lifesciences. All other authors reported no financial interests.

## Footnotes

To access the supplemental material accompanying this article, visit the online version of the *Journal of the Society for Cardiovascular Angiography & Interventions* at 10.1016/j.jscai.2026.105625.
