# Supplementary material for: Percutaneous Left Atrial Appendage Occlusion Versus Oral Anticoagulation in Atrial Fibrillation: A Systematic Review and Bayesian Meta-analysis
Source: J Soc Cardiovasc Angiogr Interv. 2026 Sep 10;5(10):105625. doi: 10.1016/j.jscai.2026.105625 (PMC13700046; doi:10.1016/j.jscai.2026.105625)
Supplement: Supplementary file 1 [file mmc1.docx]

**Search Terms**

**Cochrane CENTRAL Search Strategy:**

The search combined two concept groups: Group 1 (Left Atrial Appendage Intervention): MeSH descriptor: [Atrial Appendage] explode all trees OR ("left atrial appendage" OR "atrial appendage"):ti,ab,kw OR (LAAO OR LAAC OR "LAA closure" OR "LAA occlusion"):ti,ab,kw OR ("appendage closure" OR "appendage occlusion" OR "appendage exclusion"):ti,ab,kw OR (Watchman OR "Watchman FLX"):ti,ab,kw OR (Amulet OR "AMPLATZER Amulet" OR LAmbre OR Ultraseal OR Wavecrest):ti,ab,kw; Group 2 (Atrial Fibrillation and Anticoagulation): MeSH descriptor: [Atrial Fibrillation] explode all trees OR ("atrial fibrillation" OR "atrial flutter"):ti,ab,kw OR MeSH descriptor: [Anticoagulants] explode all trees OR (anticoagulat* OR "oral anticoagulat*"):ti,ab,kw OR (warfarin OR coumarin OR "vitamin K antagonist*"):ti,ab,kw OR (DOAC OR NOAC OR "direct oral anticoagulant*" OR "novel oral anticoagulant*"):ti,ab,kw OR (apixaban OR rivaroxaban OR dabigatran OR edoxaban):ti,ab,kw OR ("stroke prevention" OR "thromboembolism prevention" OR "embolism prevention"):ti,ab,kw.

**Web of Science Search Strategy:**

("left atrial appendage" OR "atrial appendage" OR "LAAO OR LAAC OR "LAA closure" OR "LAA occlusion" OR "appendage closure" OR "appendage occlusion" OR "appendage exclusion" OR Watchman" OR "Watchman FLX" OR "Amulet OR "AMPLATZER Amulet" OR LAmbre OR Ultraseal OR Wavecrest") AND ("atrial fibrillation" OR "atrial flutter" OR "anticoagulat* OR "oral anticoagulat*" OR "warfarin OR coumarin OR "vitamin K antagonist*" OR "DOAC" OR "NOAC" OR "direct oral anticoagulant*" OR "novel oral anticoagulant*" OR "apixaban" OR "rivaroxaban" OR "dabigatran" OR "edoxaban" OR "stroke prevention" OR "thromboembolism prevention" OR "embolism prevention") AND ("randomized" OR "randomised" OR "random* allocat*" OR "random* assign*" OR "controlled trial" OR "clinical trial" OR "noninferiority" OR "non-inferiority" OR "noninferior OR "non-inferior")

**MEDLINE (Ovid) Search Strategy:**

The search combined Group 1 (Left Atrial Appendage Intervention): exp Atrial Appendage/ OR ("left atrial appendage" or "atrial appendage").ti,ab,kw. OR (LAAO or LAAC or "LAA closure" or "LAA occlusion").ti,ab,kw. OR ("appendage closure" or "appendage occlusion" or "appendage exclusion").ti,ab,kw. OR (Watchman or "Watchman FLX").ti,ab,kw. OR (Amulet or "AMPLATZER Amulet" or LAmbre or Ultraseal or Wavecrest).ti,ab,kw.; Group 2 (Atrial Fibrillation and Anticoagulation): exp Atrial Fibrillation/ OR ("atrial fibrillation" or "atrial flutter").ti,ab,kw. OR exp Anticoagulants/ OR (anticoagulat* or "oral anticoagulat*").ti,ab,kw. OR (warfarin or coumarin or "vitamin K antagonist*").ti,ab,kw. OR (DOAC or NOAC or "direct oral anticoagulant*" or "novel oral anticoagulant*").ti,ab,kw. OR (apixaban or rivaroxaban or dabigatran or edoxaban).ti,ab,kw. OR ("stroke prevention" or "thromboembolism prevention" or "embolism prevention").ti,ab,kw.; Group 3 (Randomised Controlled Trials Filter): randomized controlled trial.pt. OR controlled clinical trial.pt. OR (randomized or randomised).ti,ab,kw. OR ("random* allocat*" or "random* assign*").ti,ab,kw. OR (controlled adj3 trial).ti,ab,kw. OR ("clinical trial" or "clinical study").ti,ab,kw. OR (noninferiority or "non-inferiority" or noninferior or "non-inferior").ti,ab,kw.

**Scopus Search Strategy:**

The search combined Group 1 (Left Atrial Appendage Intervention): TITLE-ABS-KEY("left atrial appendage" OR "atrial appendage") OR TITLE-ABS-KEY(LAAO OR LAAC OR "LAA closure" OR "LAA occlusion") OR TITLE-ABS-KEY("appendage closure" OR "appendage occlusion" OR "appendage exclusion") OR TITLE-ABS-KEY(Watchman OR "Watchman FLX") OR TITLE-ABS-KEY(Amulet OR "AMPLATZER Amulet" OR LAmbre OR Ultraseal OR Wavecrest); Group 2 (Atrial Fibrillation and Anticoagulation): TITLE-ABS-KEY("atrial fibrillation" OR "atrial flutter") OR TITLE-ABS-KEY(anticoagulat* OR "oral anticoagulat*") OR TITLE-ABS-KEY(warfarin OR coumarin OR "vitamin K antagonist*") OR TITLE-ABS-KEY(DOAC OR NOAC OR "direct oral anticoagulant*" OR "novel oral anticoagulant*") OR TITLE-ABS-KEY(apixaban OR rivaroxaban OR dabigatran OR edoxaban) OR TITLE-ABS-KEY("stroke prevention" OR "thromboembolism prevention" OR "embolism prevention"); Group 3 (Randomised Controlled Trials Filter): TITLE-ABS-KEY(randomized OR randomised) OR TITLE-ABS-KEY("random* allocat*" OR "random* assign*") OR TITLE-ABS-KEY("controlled trial" OR "clinical trial") OR TITLE-ABS-KEY(noninferiority OR "non-inferiority" OR noninferior OR "non-inferior").

**Supplementary Table S1: Baseline and Clinical Characteristics**

| **Characteristic** | **Trial** | **LAAO** | **OAC** |
| --- | --- | --- | --- |
| **Total Patients (N)** | PROTECT AF | 463 | 244 |
|  | PREVAIL | 269 | 138 |
|  | PRAGUE-17 | 201 | 201 |
|  | OPTION | 803 | 797 |
|  | CLOSURE-AF | 446 | 442 |
|  | CHAMPION-AF | 1499 | 1501 |
|  | **Total** | **3681** | **3323** |
| **Age (years, Mean ± SD)** | PROTECT AF | 71.7 ± 8.8 | 72.7 ± 9.2 |
|  | PREVAIL | 74.0 ± 7.4 | 74.9 ± 7.2 |
|  | PRAGUE-17 | 73.4 ± 6.7 | 73.2 ± 7.2 |
|  | OPTION | 69.7 ± 7.4 | 69.4 ± 7.9 |
|  | CLOSURE-AF | 78.5 ± 6.8 | 77.3 ± 7.3 |
|  | CHAMPION-AF | 71.6 ± 7.5 | 71.8 ± 7.5 |
|  | **Overall** | **72.3 ± 8.0** | **72.2 ± 8.0** |
| **Female (n, %)** | PROTECT AF | 137 (29.6%) | 73 (29.9%) |
|  | PREVAIL | 87 (32.3%) | 35 (25.4%) |
|  | PRAGUE-17 | 67 (33.3%) | 71 (35.3%) |
|  | OPTION | 283 (35.2%) | 263 (33.0%) |
|  | CLOSURE-AF | 172 (38.6%) | 171 (38.7%) |
|  | CHAMPION-AF | 485 (32.4%) | 473 (31.5%) |
|  | **Overall** | **1231 (33.4%)** | **1086 (32.7%)** |
| **BMI (kg/m², Mean ± SD)†** | PROTECT AF | 31.6 ± 6.0 | 31.3 ± 6.2 |
|  | PREVAIL | NA | NA |
|  | PRAGUE-17 | NA | NA |
|  | OPTION | 30.7 ± 5.8 | 30.7 ± 6.1 |
|  | CLOSURE-AF | 27.6 ± 5.6 | 28.1 ± 6.9 |
|  | CHAMPION-AF | NA | NA |
|  | **Overall** | **30.1 ± 6.0** | **30.0 ± 6.5** |
| **Paroxysmal AF (n, %)‡** | PROTECT AF | 200 (43.2%) | 99 (40.6%) |
|  | PREVAIL | 131 (48.7%) | 71 (51.4%) |
|  | PRAGUE-17 | 53 (26.4%) | 67 (33.3%) |
|  | OPTION | 477 (59.4%) | 501 (62.9%) |
|  | CLOSURE-AF | NA | NA |
|  | CHAMPION-AF | 1038 (69.2%) | 1028 (68.5%) |
|  | **Overall** | **1899 (58.7%)** | **1766 (61.3%)** |
| **Persistent/Permanent AF (n, %)‡** | PROTECT AF | 257 (55.5%) | 143 (58.6%) |
|  | PREVAIL | 134 (49.8%) | 67 (48.6%) |
|  | PRAGUE-17 | 148 (73.6%) | 134 (66.7%) |
|  | OPTION | 326 (40.6%) | 296 (37.1%) |
|  | CLOSURE-AF | NA | NA |
|  | CHAMPION-AF | 460 (30.7%) | 473 (31.5%) |
|  | **Overall** | **1325 (41.0%)** | **1113 (38.6%)** |
| **CHA₂DS₂-VASc (Mean ± SD)§** | PROTECT AF | NA | NA |
|  | PREVAIL | 3.8 ± 1.2 | 3.9 ± 1.2 |
|  | PRAGUE-17 | 4.7 ± 1.5 | 4.7 ± 1.5 |
|  | OPTION | 3.5 ± 1.3 | 3.5 ± 1.3 |
|  | CLOSURE-AF | 5.2 ± 1.5 | 5.1 ± 1.6 |
|  | CHAMPION-AF | 3.5 ± 1.2 | 3.5 ± 1.3 |
|  | **Overall** | **3.8 ± 1.4** | **3.8 ± 1.5** |
| **HAS-BLED (Mean ± SD)¶** | PROTECT AF | NA | NA |
|  | PREVAIL | NA | NA |
|  | PRAGUE-17 | 3.1 ± 0.9 | 3.0 ± 0.9 |
|  | OPTION | 1.2 ± 0.8 | 1.2 ± 0.8 |
|  | CLOSURE-AF | 3.1 ± 0.9 | 3.0 ± 0.9 |
|  | CHAMPION-AF | 1.3 ± 0.8 | 1.3 ± 0.8 |
|  | **Overall** | **1.7 ± 1.1** | **1.6 ± 1.1** |
| **Prior Stroke/TIA (n, %)** | PROTECT AF | 82 (17.7%) | 49 (20.1%) |
|  | PREVAIL | 74 (27.5%) | 39 (28.3%) |
|  | PRAGUE-17 | 73 (36.3%) | 69 (34.3%) |
|  | OPTION | 80 (10.0%) | 92 (11.5%) |
|  | CLOSURE-AF | 142 (31.8%) | 151 (34.2%) |
|  | CHAMPION-AF | 119 (7.9%) | 114 (7.6%) |
|  | **Overall** | **570 (15.5%)** | **514 (15.5%)** |
| **Heart Failure (n, %)#** | PROTECT AF | 124 (26.8%) | 66 (27.0%) |
|  | PREVAIL | 63 (23.4%) | 32 (23.2%) |
|  | PRAGUE-17 | 88 (43.8%) | 90 (44.8%) |
|  | OPTION | 161 (20.0%) | 166 (20.8%) |
|  | CLOSURE-AF | NA | NA |
|  | CHAMPION-AF | NA | NA |
|  | **Overall** | **436 (25.1%)** | **354 (25.7%)** |
| **Hypertension (n, %)*** | PROTECT AF | 415 (89.6%) | 220 (90.2%) |
|  | PREVAIL | 238 (88.5%) | 134 (97.1%) |
|  | PRAGUE-17 | 186 (92.5%) | 186 (92.5%) |
|  | OPTION | 721 (89.8%) | 701 (87.9%) |
|  | CLOSURE-AF | 417 (93.5%) | 417 (94.3%) |
|  | CHAMPION-AF | NA | NA |
|  | **Overall** | **1977 (90.6%)** | **1658 (91.0%)** |
| **Diabetes Mellitus (n, %)*** | PROTECT AF | 113 (24.4%) | 72 (29.5%) |
|  | PREVAIL | 91 (33.8%) | 41 (29.7%) |
|  | PRAGUE-17 | 73 (36.3%) | 90 (44.8%) |
|  | OPTION | 222 (27.6%) | 221 (27.7%) |
|  | CLOSURE-AF | 175 (39.2%) | 186 (42.1%) |
|  | CHAMPION-AF | NA | NA |
|  | **Overall** | **674 (30.9%)** | **610 (33.5%)** |
| **Prior MI (n, %)**** | PROTECT AF | NA | NA |
|  | PREVAIL | NA | NA |
|  | PRAGUE-17 | 30 (14.9%) | 39 (19.4%) |
|  | OPTION | NA | NA |
|  | CLOSURE-AF | 115 (25.8%) | 89 (20.1%) |
|  | CHAMPION-AF | NA | NA |
|  | **Overall** | **145 (22.4%)** | **128 (19.9%)** |
| **CKD, eGFR <60 (n, %)‡‡** | PROTECT AF | NA | NA |
|  | PREVAIL | NA | NA |
|  | PRAGUE-17 | NA | NA |
|  | OPTION | NA | NA |
|  | CLOSURE-AF | 264 (59.2%) | 246 (55.7%) |
|  | CHAMPION-AF | NA | NA |
|  | **Overall** | **264 (59.2%)** | **246 (55.7%)** |
| **Prior Major Bleeding (n, %)‡‡** | PROTECT AF | NA | NA |
|  | PREVAIL | NA | NA |
|  | PRAGUE-17 | NA | NA |
|  | OPTION | NA | NA |
|  | CLOSURE-AF | 188 (42.2%) | 191 (43.2%) |
|  | CHAMPION-AF | NA | NA |
|  | **Overall** | **188 (42.2%)** | **191 (43.2%)** |
| **LVEF (%, Mean ± SD)§§** | PROTECT AF | 57.3 ± 9.7 | 56.7 ± 10.1 |
|  | PREVAIL | 55.4 ± 10.0 | 56.0 ± 9.8 |
|  | PRAGUE-17 | NA | NA |
|  | OPTION | NA | NA |
|  | CLOSURE-AF | NA | NA |
|  | CHAMPION-AF | NA | NA |
|  | **Overall** | **56.6 ± 9.9** | **56.5 ± 10.0** |

Data presented as Mean ± SD or n (%). LAAO = Left Atrial Appendage Occlusion. OAC = Oral Anticoagulation. NA = Not available. AF = Atrial Fibrillation. BMI = Body Mass Index. CKD = Chronic Kidney Disease. eGFR = estimated Glomerular Filtration Rate. LVEF = Left Ventricular Ejection Fraction. MI = Myocardial Infarction. TIA = Transient Ischemic Attack.

**^†^**BMI: PROTECT AF, OPTION, CLOSURE-AF.

**^‡^**Paroxysmal and Persistent/Permanent AF: all trials except CLOSURE-AF.

**^§^**CHA₂DS₂-VASc: all trials except PROTECT AF.

**^¶^**HAS-BLED: PRAGUE-17, OPTION, CLOSURE-AF, CHAMPION-AF.

**^#^**Heart Failure: PROTECT AF, PREVAIL, PRAGUE-17, OPTION.

**^*^**Hypertension and Diabetes Mellitus: all trials except CHAMPION-AF.

**^**^**Prior MI: PRAGUE-17, CLOSURE-AF.

**^‡‡^**CKD and Prior Major Bleeding: CLOSURE-AF only.

**^§§^**LVEF: PROTECT AF, PREVAIL.

**Supplementary Figure S1.** Risk of bias assessment of included trials (RoB-2 tool). Domains: randomization process; deviations from intended interventions; missing outcome data; outcome measurement; selection of reported results.

**Supplementary Figure S2. Sequential Bayesian updating of the ischemic stroke incidence rate ratio.**

Each curve represents the posterior distribution after adding one trial in chronological order, beginning with a vague prior (Normal (0, 10²) on the log-IRR). Trials enter in the order: PROTECT AF, PREVAIL, PRAGUE-17, OPTION, CLOSURE-AF, CHAMPION-AF. Progressive narrowing of successive curves reflects accumulating evidence; the persistent shift to the right of unity reflects the consistent direction of effect across trials. The sequential view shows that the posterior crosses to the right of unity once OPTION and the contemporary DOAC trials enter, with the central tendency concentrating between IRR 1.0 and 1.3 by the time CHAMPION-AF is added. The width of successive curves contracts as the cumulative event count grows.

**Supplementary Figure S3: Bidirectional posterior probability for the any-stroke composite under LAAO versus OAC.**

The same posterior distribution is summarized under two conventions. The red curve plots the harm convention: posterior probability that LAAO increases the annual any-stroke rate by at least x percentage points. The blue curve plots the benefit convention: posterior probability that LAAO decreases the annual any-stroke rate by at least x percentage points. At x = 0 the two probabilities sum to approximately 1 (the residual mass at exact equality is negligible). Both conventions are presented because any stroke is a patient-centered composite for which neither direction of effect is a priori expected. Reading the curves together gives the full directional summary: where the harm curve sits high relative to the benefit curve, the data favor harm at that magnitude. Method: Bayesian hierarchical Poisson model with a vague prior (Normal (0, 10²) on the log-IRR); 200,000 post-warmup draws. Absolute rate differences are computed relative to the pooled OAC any-stroke baseline rate of 1.08%/yr. Reference points are marked at thresholds of 0.5, 1.0, and 1.5 pp/year.

**Trials included**: PROTECT AF, PREVAIL, PRAGUE-17, OPTION, CLOSURE-AF, and CHAMPION-AF. Any-stroke counts include both ischemic and hemorrhagic events; PREVAIL hemorrhagic counts (1 LAAO vs 0 warfarin) are from Holmes et al., JACC 2014, Table 3.

**Abbreviations**: LAAO, left atrial appendage occlusion; OAC, oral anticoagulation; pp, percentage points.
